# Bending effects of ZnO nanorod metal–semiconductor–metal photodetectors on flexible polyimide substrate

**DOI:** 10.1186/1556-276X-7-214

**Published:** 2012-04-12

**Authors:** Tse-Pu Chen, Sheng-Joue Young, Shoou-Jinn Chang, Chih-Hung Hsiao, Yu-Jung Hsu

**Affiliations:** 1 Institute of Microelectronics and Department of Electrical Engineering, Center for Micro/Nano Science and Technology, Advanced Optoelectronic Technology Center, National Cheng Kung University, Tainan, 701, Taiwan; 2 Department of Electronic Engineering, National Formosa University, Yunlin, 632, Taiwan; 3 Institute of Electro-Optical Science and Engineering, National Cheng Kung University, Tainan, 701, Taiwan

**Keywords:** ZnO, Nanorod, MSM, Photodetector, Flexible

## Abstract

The authors report the fabrication and I-V characteristics of ZnO nanorod metal–semiconductor–metal photodetectors on flexible polyimide substrate. From field-emission scanning electron microscopy and X-ray diffraction spectrum, ZnO nanorods had a (0002) crystal orientation and a wurtzite hexagonal structure. During the I-V and response measurement, the flexible substrates were measured with (i.e., the radius of curvatures was 0.2 cm) and without bending. From I-V results, the dark current decreased, and the UV-to-visible rejection ratio increased slightly in bending situation. The decreasing tendency of the dark current under bending condition may be attributed to the increase of the Schottky barrier height.

## Background

Zinc oxide (ZnO), a nanostructured material that has been widely investigated, has a wide energy band gap of 3.37 eV at room temperature, high optical gain of 300/cm which is higher than that of GaN (100/cm) [1], and large exciton binding energy of 60 meV [2] which is higher than that of ZnSe (22 meV) and GaN (25 meV). The large exciton binding energy provides high-luminescence efficiency of light emission at or above room temperature. ZnO has slightly higher saturation velocity of 3.2 × 10^7^ cm/s [3] than GaN, InGaN, and AlGaN [4,5], but the room temperature electron Hall mobility (205 cm^2^/V/s/) [6] is lower than that of GaN. ZnO has high mechanical and thermal stabilities, and radiation hardness for devices used in nuclear and space applications. ZnO also has lower growth temperature and material cost than III-nitride materials.

According to the researches, GaN and its alloys with AlN and InN cover the spectral range from red to vacuum UV (1.9 to 6.2 eV). Therefore, III-nitride materials have attracted a great deal of attention since the commercialization of light-emitting diodes. Not only GaN but also ZnO can tune the value of band gap by forming the ternary alloy of ZnMgO and ZnCdO with MgO and CdO [7], respectively.

Over the past decade, ZnO-based and III-nitride-based light-emitting diodes (LEDs) and laser diodes (LDs) have attracted much interest for display, illumination, and mobile phone backlights. Recently, Zhang et al. [8] studied on gain properties of high Al-content AlGaN-delta-GaN quantum wells (QWs) for mid- and deep-UV lasers. Zhao et al. [9] investigated the QW structures with large overlap design to enhance the internal quantum efficiency for InGaN QW-based LEDs. Shukla [10] studied a p-n junction LED employing ZnO/MgZnO QW active layer on a *c*-plane sapphire by the pulsed-laser deposition technique. Ahn et al. [11] showed p-n heterojunction LEDs that were formed from a p-Si thin film/nanostructured n-ZnO by a dielectrophoresis method. Except for the LEDs and LDs, ZnO makes it as a promising functional material for the electronic device manufacture such as field emission, photodetectors (PD), solar cells, waveguides, and chemical or biosensors.

To date, many groups have reported encouraging results for ZnO-based photodetectors. It is important that ZnO-based PDs be used in various military and commercial applications, like missile launching and flame detections, optical communications, and ozone layer monitoring. Indeed, ZnO-based PDs have various types, such as p-n junction PDs, p-i-n PDs, Schottky barrier PDs, and metal–semiconductor–metal (MSM) PDs [12-15]. Among the above structures, the MSM structure is a practical application due to its easy fabrication, low dark current and device noise values, high-response speed, and compatibility with integrated circuit technology. Recently, Yen et al. [16] obtained high photocurrent generation with ZnO/Si heterostructure MSM PDs by an avalanche multiplication in the ZnO layer. GaN-based MSM PDs also can detect the UV region, but a significant number of threading dislocations exit in GaN epilayers due to the large mismatches in lattice constant and thermal expansion coefficient between GaN and substrate, like sapphire and Si. Recently, Li et al. [17] found that dislocations had strong influence on the dark current and responsivity of the PDs. Consequently, ZnO-based materials have more advantages for PDs than GaN-based materials.

Recently, various one-dimensional (1-D) ZnO semiconducting nanostructures have been synthesized like nanorods, nanotubes, and nanobelts [18-20]. Most importantly, ZnO is well known as a piezoelectric material. Because of semiconductor properties and the coupling of piezoelectric of ZnO, many groups extensively investigated nanogenerators, piezoelectric field effect transistors, and piezoelectric strain and piezoelectric humidity/chemical sensors [21-24].

Today, many groups investigate the optoelectronic devices grown on flexible substrates, but the problem of these flexible substrates is that they must use low-temperature process. ZnO is one of the most suitable materials on flexible substrate because it can be prepared by low temperature processes, like sputter and aqueous method. Growth of 1-D ZnO semiconducting nanostructures on flexible substrates has been widely reported which are ideal candidates for LEDs, solar cell, file-effect transistors, personal health monitors, and field emission [22,25-27] due to cheap, lightweight, and portable characteristics. However, the mechanical bending characteristics of the ZnO nanorod MSM structures fabricated on flexible substrates have rarely been investigated.

In this letter, we fabricated MSM ultraviolet PDs with ZnO nanorods on flexible substrate by aqueous method. The advantages of aqueous method are low cost, simple process, low temperature, and high product yield. Further, the fabricated devices were measured with and without strong mechanical bending. The optical and electric properties and the working principle of the ZnO nanorod MSM PDs would be presented in detail.

## Methods

The polyimide (PI) substrates were ultrasonically cleaned in methanol, isopropanol, and deionized water and dried with nitrogen. ZnO-seed layers with a thickness of about 130 nm were grown on polyimide substrates in a low-pressure RF sputter reactor. In our sputtering system, the background pressure in the sputtering chamber was evacuated to about 8 × 10^−6^ Torr with a turbo pump. The deposition processes were performed in Ar and O_2_ ambient at an applied RF power of 120 W. After deposition, the as-grown samples were annealed 40 min in oxygen flow at 300°C in a quartz tube. After annealing, the samples were cooled to room temperature naturally, and MSM PDs were subsequently fabricated on the annealed samples. The ZnO PDs were fabricated using interdigital MSM structures by standard lithography. Using e-beam evaporation, Ag/Au (40/50 nm) contact electrodes were deposited on the samples served as Schottky contacts. The metal contact did not anneal by furnace or RTA. Then, we used photoresist to protect the electrodes by standard lithography. ZnO nanorod arrays were grown using an aqueous solution containing zinc nitrate (Zn(NO_3_)_2_) and hexamethylenetetramine (HMTA) for 1 h at 90°C. During ZnO nanorod synthesis, Zn(NO_3_)_2_ and HMTA act as a Zn^2+^ source and a pH buffer, respectively, which keep the pH value at a constant [28]. After the process of the grown ZnO nanorods, the photoresist was lift-off, and there is no ZnO film or short nanorod formed on the metal electrode.

**Figure 1:**
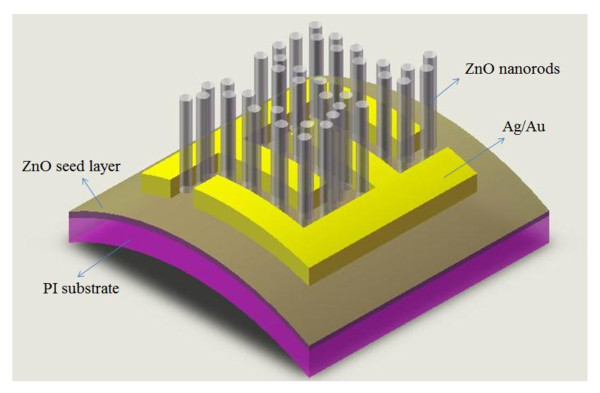
Schematic of the ZnO nanorod MSM PD prepared on flexible substrate.

Figure 1 shows the schematic of the proposed ZnO nanorod MSM photodetector. The morphologies and size distribution of the ZnO nanorod PDs were characterized by field-emission scanning electron microscopy (JSM-7000 F, JEOL Ltd., Akishima, Tokyo, Japan) which was operated at 10 keV. X-ray diffraction (XRD) measurement was then utilized to characterize the optical and crystallographic properties of the as-grown ZnO nanorods. An HP-4156 C semiconductor parameter analyzer (Agilent Technologies Inc., Santa Clara, CA, USA) was then employed to measure current–voltage (I-V) characteristics of the proposed ZnO nanorod MSM photodetector. The photo-responsivity was obtained using the TRIAX 180 system (HORIBA Ltd., Minami-Ku, Kyoto, Japan) with a 300 W xenon arc lamp light source and standard synchronous detection scheme.

## Results and discussion

**Figure 2:**
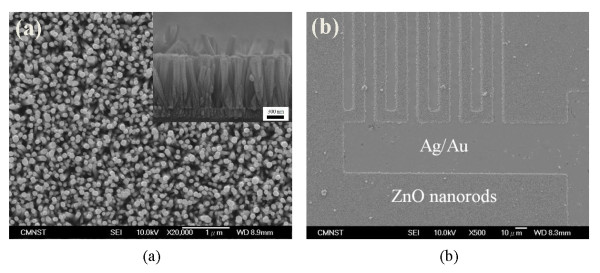
**SEM images.** (**a**) Top view of the ZnO nanorods. Inset: the cross-sectional image of the nanorods. (**b**) Top view of the ZnO nanorods MSM photodetector.

Figure 2a and its inset shows the top view and cross-sectional SEM images of the ZnO nanorods grown on the flexible substrate by aqueous method. From Figure 2a, a large of ZnO nanorod array grew uniformly on the flexible substrate with varying diameter of nanorods (80–100 nm). The shapes of the nanorods had wurtzite structure. The inset of the Figure 2a shows the cross-section of the nanorods. As seen, most of the nanorods were well aligned, perpendicular to the substrate which the length of the nanorods can reach up to approximately 1 μm. Figure 2b shows the top view of the ZnO MSM nanorod device. From the image, it can be seen that the ZnO nanorod arrays selectively distributed among the interdigitated electrodes of the device. It confirmed that the electrodes can effectively be protected by the photoresist, which avoid the nanorods growing onto the electrodes. Therefore, our method can fabricate MSM PDs with a large area aligned ZnO nanorods to enhance the performance.

**Figure 3:**
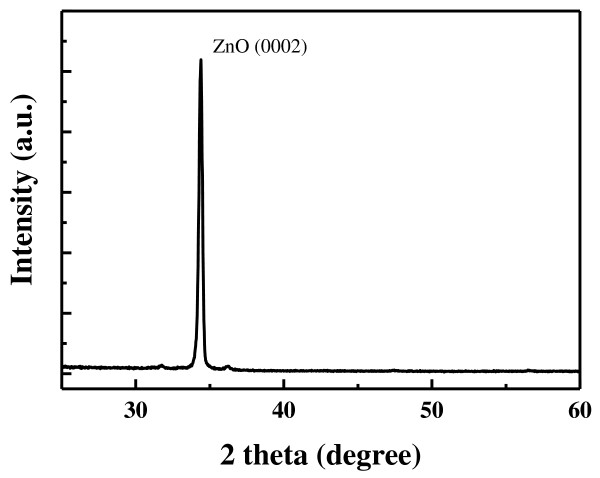
XRD spectrum measured from vertically aligned ZnO nanorods.

Figure 3 shows the typical X-ray diffraction spectrum of the ZnO nanorods prepared on the ZnO/PI substrate. According to XRD result, it depicts the diffraction angles of 34.4° assigned as ZnO (0002) reflection. The strong (0002) reflection with narrow width indicates that the ZnO nanorods were crystallized in wurtzite structure and preferentially grown along the *c*-axis direction.

**Figure 4:**
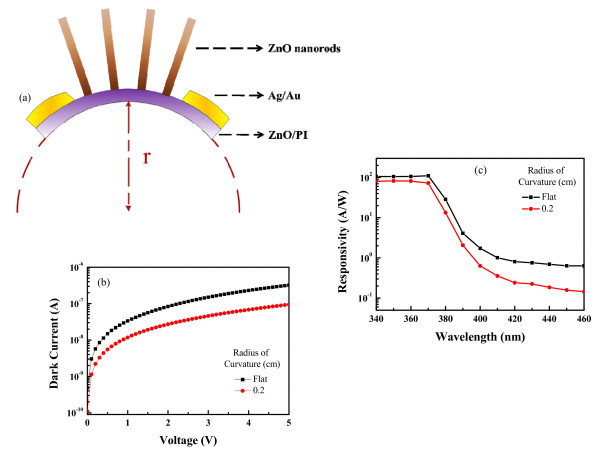
**Schematic drawing of the bending device, and the dark I-V characteristics and responsivity characteristics.** (**a**) Schematic drawing of the bending device. (**b**) The I-V and (**c**) responsivity characteristics of the PD measured from flat and bending substrate in the dark.

The ZnO nanorod MSM PDs were fabricated on flexible polyimide substrate, so the performance under bending conditions is of major importance. It is important to understand the characteristics of the flexible ZnO nanorod MSM PDs with bending and without bending for the future of plastic electronic productions. Stable performance at bending situation is a critical challenge. For this reason, we measured the electrical characteristics of the PDs with bending and without bending. Before the measurement, we prepared a holder with semicircular shape (the radius of the holder was 0.2 cm) and fixed the ZnO nanorod MSM PDs on it, which could make the device bending. For convenience of realization, Figure 4a shows a schematic drawing of the bending device, where *r* corresponds to the radius of the curvature of bending. A smaller radius of the curvature of bending means increased bending. Figure 4b shows the current–voltage characteristics of ZnO nanorod PDs under flat and 0.2-cm radius of bending curvature, respectively. From the result, it could be known that dark current decreased slightly under bending, but the shift was lesser than one order of magnitude. The I-V behavior of the device was modulated due to the change in Schottky barrier height (SBH) at the metal–semiconductor interface. It is well known that the lacking of center symmetry in ZnO lead ionic polarization can be induced by strain [29]. When the ZnO nanorod PDs are bent by an external force, the potential is induced by the piezoelectric effect which is created by the relative displacement of the Zn^2+^ with respect to the O^2−^, and these piezoelectric ionic charges cannot move freely until external force releases [21,30]. These piezoelectric ionic charges could affect the charge transport strongly. The changing of piezopotential would shift the local Fermi level and modify the local conduction band structure. Therefore, the PDs under tensile strain would increase the SBH and influence the charge transport property by the piezoelectric effect and the band structure.

In our experiment, the structure of our device is MSM structure which is like two back-to-back Schottky diodes, and our devices were measured at room temperature and the ZnO nanorods had low doping. Therefore, the current transport mechanism followed the thermionic emission-diffusion theory (for *V* > > 3 kT/q, approximately 77 mV) [31]. According to the literature [23], the thermionic emission-diffusion theory can be simplified by assuming some parameters are independent of strain, and then the change of SBH can be determined by the equation below [23]:

(1) $$\ln\left[I\left({\text{ϵ}}_{\text{zz}}\right)/I\left(0\right)\right]=-\text{Δ}{\text{ϕ}}_{\text{s}}/\text{kT}$$

where *I* (ϵ_zz_) and *I*(0) are the current measured from the ZnO nanorod MSM PDs at a fixed bias with and without being strained, respectively. After calculation, the changes of SBH were 27 meV and 29 meV for two biases of 1 and 2 V, respectively. From the two values, it can be known that the change of the SBH is not very sensitive to the bias applied across the device.

Owing to the outward bending of the substrate, piezoelectric effects could be induced and it could be attributed to the tensile strain between n-ZnO lattices. As we know, strain can be used in many applications. In microelectronic industry, scaling of MOSFETs has attracted many people to study it for the improvement in integrated circuit density and performance. However, MOSFET size reduction has met technological challenges such as short-channel effect and high leakage current. Therefore, strain has been an increase in interest in semiconductor material since the 1950s. Using appropriate tensile and compressive strain components in the n- and p-MOSFET's channel region could enhance its performance [32-35].

Figure 4c shows the responsivity characteristics of the ZnO nanorod PDs under flat and bending curvature. In this figure, the responsivities in visible wavelengths decreased slightly under bending, and the cutoff wavelength was the same. The UV-to-visible rejection ratios, defined as the ratio of the responsivity at 370 nm and at 460 nm, are 174.6 and 503.2 for flat and 0.2-cm radius of curvature bending, respectively. The rejection ratio of the 0.2-cm radius of curvature bending had a larger value that could be attributed to the smaller dark current.

From the responsivity characteristics of the ZnO nanorod PDs, the PDs had high responsivities under UV-light illumination. In addition, the photoconductive gain is given by the following equation [36]:

(2) G=Rhv/qη

where *G* is the internal gain, *R* is the responsivity, *h* is the Plank's constant, *v* is the frequency of the light, *q* is the electronic charge, and *η* is the quantum efficiency. Assuming *η* is 1 for simplicity, the internal gains of the ZnO nanorod PDs measured at flat and 0.2-cm radius of curvature bending were 3.71 × 10^2^ and 2.44 × 10^2^, respectively. Both PDs had high responsivities due to ZnO nanorod large surface-to-volume ratio and the existence of the oxygen-related hole surface states on the ZnO nanorods surface. The oxygen molecules can adsorb on the ZnO nanorod surface by capturing free electrons and desorb from the surface by illustrating UV light which lead to an increase in the free carrier concentration, a decrease in the width of the depletion layer, and a reduction of the Schottky barrier height [37]. Recently, Soci et al. [37] published the ZnO nanowire PDs with high internal gain. They fabricated a ZnO photodetector with one ZnO nanowire. Their device has achieved an internal gain of approximately 10^8^. Instead, our device has smaller internal gain than theirs. This could be attributed to the high density of nanorod array of our device and there were small gaps between the freestanding nanorods. When the nanorods were illustrated by the UV light, the oxygen molecules desorbed from the nanorods surface. However, the nanorods were too close that make the oxygen molecules quickly readsorb with the nanorod surface by capturing free electrons and decrease the photocurrent. Thus, the internal gain of our device was smaller than theirs.

## Conclusions

In summary, we reported the fabrication and I-V characteristics of ZnO nanorod PDs on PI substrate. The crystal and optical properties of ZnO nanorods had been investigated by SEM and XRD. They showed the ZnO nanorods grown on the PI substrate had high density array, hexagonal wurtzite structure and well (0002) crystalline phase. From I-V curve, it was found that the dark current decreased slightly and the UV-to-visible rejection ratio increased slightly in bending situation. The decreasing tendency of dark current under bending may be attributed to the increase of the Schottky barrier height. Consequently, ZnO nanorods have great potential for high-performance UV photodetectors, and polyimide is a promising substrate for flexible PDs and devices.

## Competing interests

The authors declare that they have no competing interests.

## Authors' contributions

TPC, SJY, and SJC designed the whole experimental procedure and related analysis. CHH participated in data analysis. YJH did the nanowire growth and other experiments. All authors read and approved the final manuscript.
